# Digital dashboards for direct oral anticoagulant surveillance, intervention and operational efficiency: uptake, obstacles, and opportunities

**DOI:** 10.1007/s11239-023-02893-9

**Published:** 2023-10-15

**Authors:** Darren M. Triller, Aaron S. Wilson, Arthur L. Allen, Allison E. Burnett, Julie Ann Gouveia-Pisano, Allison Brenner, Barbara K. Pritchard, Charles Medico, Geoffrey D. Barnes

**Affiliations:** 1 Anticoagulation Forum, 17 Lincoln St, Suite 2B, Newton, MA 02461 USA; 2 University of Utah College of Pharmacy, Salt Lake City, UT 84112 USA; 3 Veterans Administration Salt Lake City Health Care System, Salt Lake City, UT USA; 4 University of New Mexico Medical Center, Albuquerque, NM USA; 5 Pfizer Inc., New York, NY USA; 6 University of Michigan Medical Center, Ann Arbor, MI USA

**Keywords:** Dashboard, Direct oral anticoagulant, Electronic health record, Population health, Stewardship

## Abstract

Direct oral anticoagulants (DOAC) are the most widely prescribed oral anticoagulants in the United States. Despite advantages over warfarin, system-level improvements are needed to optimize outcomes. While Veterans Health Administration and others have described successful DOAC management dashboard implementation, the extent of use nationally is unknown. A survey of Anticoagulation Forum’s members was conducted to assess access to digital tools available within a dashboard and to describe implementation models. An Expert Forum was subsequently convened to identify barriers to dashboard development and adoption. Responses were received from 340 targeted recipients (8.5% of invitees). Only a minority of inpatient (25/52, 48.1%) and outpatient (47/133, 35.3%) respondents outside of Veterans Health Administration were able to generate rosters of DOAC users on-demand, and fewer had the ability to digitally display key clinical data elements, identify drug-related problems, document interventions, or generate reports. The lack of regulatory requirements regarding Anticoagulation Stewardship was identified by the Expert Forum as the major barrier to widespread development of digital tools for improved anticoagulation management. While some health systems have demonstrated the feasibility of DOAC dashboards and described their impact on quality and efficiency, these tools do not appear to be widely available in the United States apart from Veterans Health Administration. The lack of regulatory requirements for Anticoagulation Stewardship may be the primary barrier to the development of digital resources to better manage anticoagulants. Efforts to secure regulatory requirements for Anticoagulation Stewardship are needed, and evidence of improvements in clinical and financial outcomes through DOAC dashboard use will likely bolster such efforts.

**Supplementary Information:**

The online version contains supplementary material available at 10.1007/s11239-023-02893-9.

## Highlights

- Digital dashboards have the potential to support high-quality anticoagulation management
- Outpatient sites from within the Veterans Health Administration (VHA) report broad adoption of an advanced digital dashboard
- Only a minority of respondents from non-VHA sites report access to advanced digital tools to support population management of anticoagulated patients
- Major electronic health record systems do not yet provide advanced features for management of anticoagulated patient populations
- The lack of regulatory mandates is perceived as a major barrier to the creation and spread of such tools
- Advances in regulatory oversite and digital tool development and dissemination are necessary to support Anticoagulation Stewardship implementation in the US

## Background

Direct oral anticoagulants (DOACs) are the most commonly prescribed oral anticoagulants in the United States, eclipsing warfarin due to their superior efficacy, enhanced safety profiles, simpler dosing regimens, and the lack of frequent laboratory monitoring requirements [1]. Despite their proven efficacy, the use of anticoagulants carries an inherent risk of bleeding. In fact, anticoagulants are the leading cause of adverse drug events in the emergency room [2]. Modifiable factors such as inappropriate prescribing, drug-drug interactions, misuse by patients, and poorly managed care transitions can contribute to serious and life-threatening bleeding events that may be preventable through improved management processes [3]. Likewise, under-prescribing, “off-label” dosing, and suboptimal patient adherence may contribute to avoidable thrombotic events, such as stroke [4, 5].

Recognizing the need to improve the quality and safety of all anticoagulant use, multiple governmental and non-governmental organizations have undertaken efforts to advance the spread and adoption of Anticoagulation Stewardship at the national level [6–10]. Confronted with the need to improve care processes for the growing number of patients utilizing DOACs while simultaneously faced with limited resources to achieve these goals, innovative health systems have begun to leverage electronic health record (EHR)-based “dashboards” to better surveille and manage populations of DOAC users while generating operational efficiencies [11–16].

The Veterans Health Administration (VHA) has long been recognized as a leader in outpatient warfarin management [9, 17, 18] and in recent years has successfully developed, implemented, and scaled a novel DOAC population management model utilizing a digital DOAC dashboard [11–16]. Originally launched in a single region in 2016, the dashboard has since been utilized in over 90% of the 164 locations nationally, demonstrating significant reductions in inappropriate DOAC dosing and significant improvements in clinician intervention efficiency. Similar DOAC dashboard implementation efforts are also underway as part of the Michigan Anticoagulation Quality Improvement Initiative (MAQI^2^), a multicenter collaboration of anticoagulation management clinics across the state of Michigan [12, 13]. These sites are currently engaged in formal research studies and data characterizing the impact of dashboard implementation on objective clinical and fiscal outcomes is forthcoming.

While these notable health systems have successfully implemented novel care models based on DOAC dashboard availability, it remains unclear whether such systems are in use by other health systems or care facilities nationally. It is also uncertain what digital features characterize the dashboards that are in use elsewhere, and what barriers impede the adoption of these proactive surveillance and intervention systems.

To address this gap in knowledge, we first conducted a scoping review of the medical literature (published separately; doi: 10.1007/s11239-023-02880-0). Informed by the initial literature search, we then conducted a survey of the Anticoagulation Forum (ACF, www.acforum.org) membership and convened a multidisciplinary expert panel to explore their visions for DOAC dashboards and identify key barriers to the advancement of such tools in clinical care settings.

## Methods

### Survey development

A digital survey consisting of 36 questions was created and disseminated to assess respondent access to digital dashboards supporting the safe and effective use of DOACs in the inpatient and outpatient settings (Supplementary file 1). It sought to characterize the ability of existing digital resources to display data fields important to DOAC management, to alert users to common DOAC-related problems, and to generate reports related to DOAC management. It also sought to characterize the maturity of any existing dashboard implementation efforts and models while evaluating current practice behaviors around population health digital tools and the opportunity for future utilization.

The survey instrument was developed using Survey Monkey (San Mateo, CA) and disseminated via email. Questions were informed by the findings of a prior scoping review of the published literature and refined iteratively through collaboration among the authors, informaticists, and an external expert with subject matter expertise in hematology and survey creation (SM, acknowledgement; literature review findings published elsewhere; doi: 10.1007/s11239-023-02880-0). While some open text comment fields were provided in the survey instrument to capture optional detail, all data included in the current analysis stemmed from structured binary (yes/no) or multiple-choice responses. All response fields required completion to progress to subsequent questions, and branch logic was utilized to minimize unnecessary and non-applicable responses based on preceding responses, as described below.

**Fig. 1:**
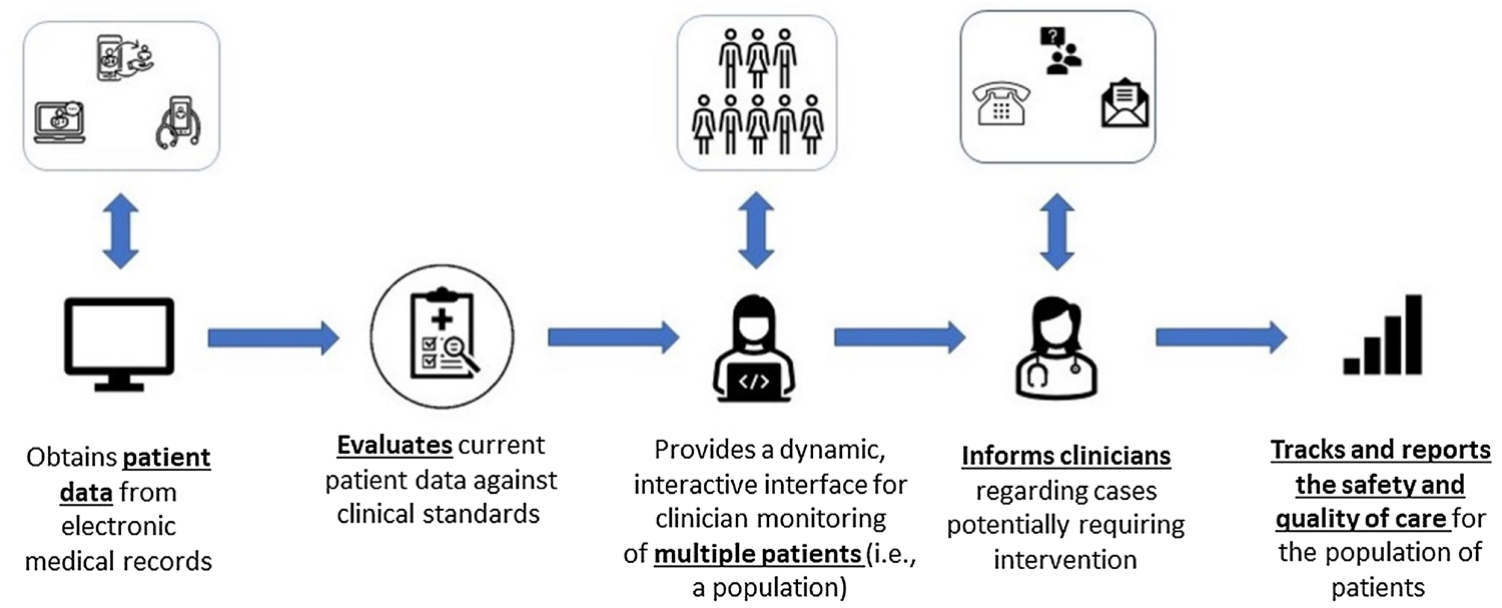
Dashboard definition. Figure based on inclusion and exclusion criteria utilized by Tsang et al. [21]; To assure clarity for respondents, this figure was accompanied by a 2 min audio narration in the digital survey

Having identified variability in the terminology used across published manuscripts, the survey introduction included an evidence-based image (Fig. 1) with audio narration describing the definition of “dashboard” for the purpose of survey completion. In a central series of questions, recipients were then specifically asked about their access to a DOAC “dashboard” in their work setting.

Because sites that were unable to generate an on-demand DOAC roster would, by definition, be unable to have access to more advanced dashboard features, the digital survey instrument utilized branch logic to only present these questions to sites with roster-generating capabilities. A “no” response was therefore imputed to all other sites for these questions and incorporated into the rates presented in the final analysis.

### Survey analysis

**Fig. 2:**
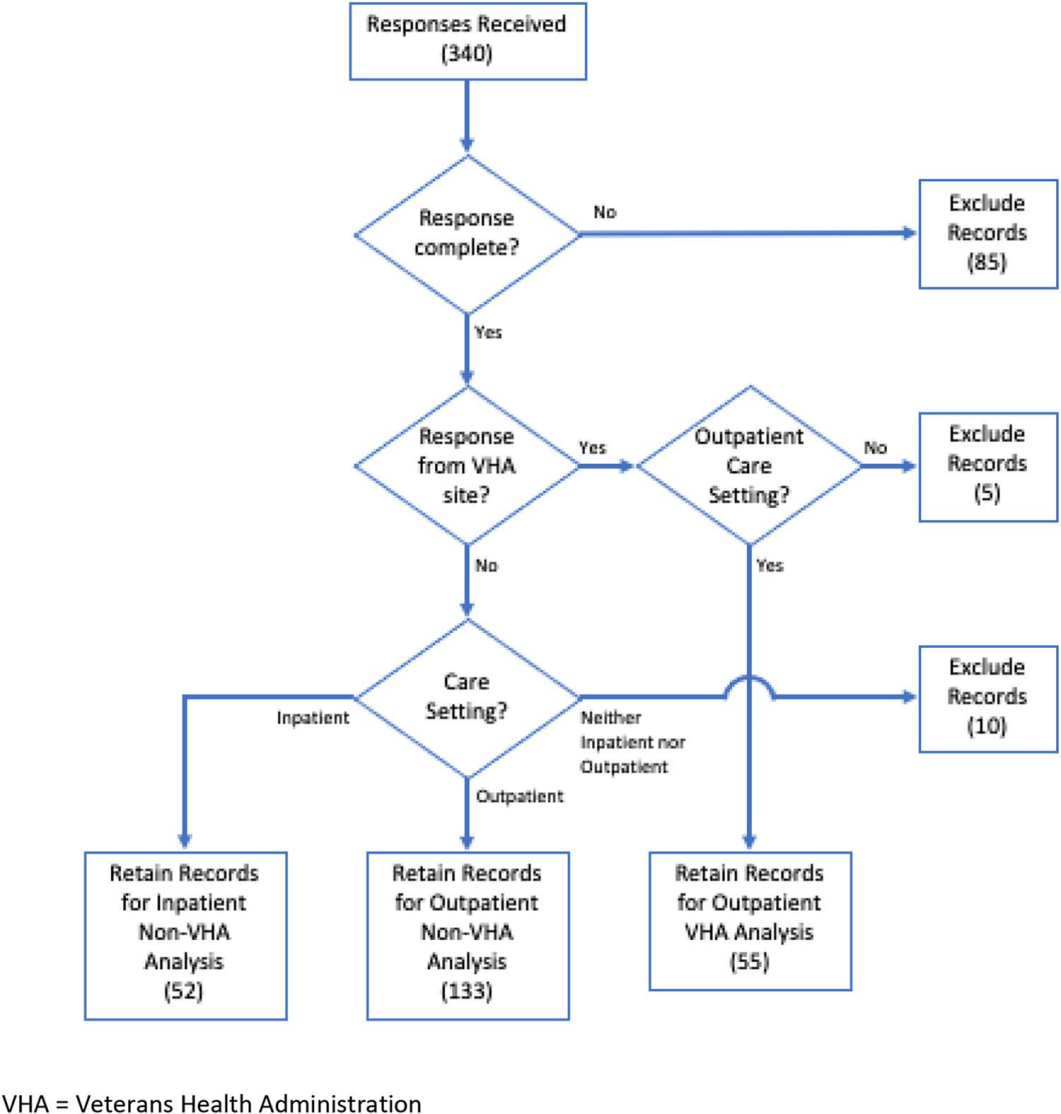
Flow diagram of survey response exclusion and inclusion process

As the prior literature evaluation showed that clinics within the VHA are known to have access to an established DOAC management dashboard [11–15], respondents from outpatient care settings were categorized as VHA or non-VHA, with statistical tests being performed to compare responses among these two groups (chi square test for categorical variables). Data analysis was performed in R (Vienna, Austria) and a p value of < 0.05 was considered to be statistically significant. No statistical tests were performed regarding the responses from acute care sites due to the small number of responses from VHA acute care sites (Fig. 2.).

## Results

### Survey respondents

The survey generated 340 total responses (8.5% response rate; Fig. 2.). Because the electronic survey utilized required fields and branch logic to promote data integrity, the failure to complete a question could have a negative cascading effect on the response rate to subsequent questions. Upon evaluation of the dataset, it was determined that records that failed to describe access to a DOAC dashboard or characterize the respondent’s care setting (VHA vs. non-VHA, inpatient vs. outpatient) would be considered incomplete and excluded from analysis (85), as the successive survey responses would be missing or of questionable value.

Of the remaining complete responses, those associated with VHA sites were then identified (60) and being that the sample from VHA acute care settings was small (5), only the responses from the outpatient setting would be retained (55). Complete responses from non-VHA sites were then evaluated by practice setting, categorized as inpatient (52) or outpatient (133), and included in the analysis (Fig. 2.).

### Respondent characteristics and work environments

Among respondents from the outpatient setting, VHA site respondents were significantly more likely to be pharmacists and to have direct patient care roles than non-VHA sites (p < 0.001; Table 1). Epic was the predominant electronic medical record system (EMR) among non-VHA sites (96/133, 72.2%; Table 2.), while nearly all VHA sites reported use of the VHA’s VistA Computerized Patient Record System (CPRS; 53/55, 96.4%, p < 0.001). VHA respondents were significantly more likely to report the use of additional software for anticoagulation management (46/55, 83.6% vs. 43/133, 32.3% respectively, p < 0.001), to have an organized service for DOAC management (54/55, 98.2% vs. 97/133, 72.9% respectively, p < 0.001), and to report having access to a DOAC management dashboard (54/55, 98.2% vs. 28/133, 21.1% respectively, p < 0.001). In contrast, non-VHA sites expressed stronger need for additional digital resources to enhance DOAC management (p = 0.018).

**Table 1:** Respondent characteristics

	Inpatient	Outpatient		
	Non-VHA (n = 52)	Non-VHA (n = 133)	VHA (n = 55)	
	Count (%)	Count (%)	Count (%)	p Value^†^
*Profession*				
Advanced practice nurse	2 (3.8%)	13 (9.8%)	0 (0.0%)	< 0.001^‡^
Pharmacist	44 (84.6%)	89 (66.9%)	54 (98.2%)	
Physician	6 (11.5%)	12 (9%)	1 (1.8%)	
Registered nurse	0 (0%)	19 (14.3%)	0 (0.0%)	
Practice type*				
Adult internal medicine	22 (42.3%)	–	–	–
Anticoagulation clinic	–	115 (86.5%)	52 (94.5%)	NS
Cardiology	14 (26.9%)	15 (11.3%)	1 (1.8%)	0.034
Critical care	15 (28.8%)	–	–	–
Emergency department	11 (21.2%)	–	–	–
Hematology	8 (15.4%)	13 (9.8%)	1 (1.8%)	NS
Neurology	6 (11.5%)	–	–	–
Surgery	12 (23.1%)	–	–	–
Stewardship	36 (69.2%)	1 (0.8%)	0 (0.0%)	NS
Anticoagulation forum center of excellence**	11 (21.2%)	65 (48.9%)	11 (20.0%)	< 0.001
*Primary role*				
Direct patient care	34 (65.4%)	95 (71.4%)	51 (92.7%)	0.029^‡^
Clinic/department management	10 (19.2%)	28 (21.1%)	4 (7.3%)	
Other	8 (15.4%)	10 (7.5%)	0 (0.0%)	

*Multiple responses possible, available response options varied by setting type

**Centers of excellence designation refers to a voluntary self-assessment program available through anticoagulation forum (https://acforum-excellence.org)

^†^Statistical tests compared outpatient groups only

^‡^Statistical test performed across group of response options

*VHA* Veterans Health Administration

**Table 2:** Descriptions of respondent work environments

	Inpatient	Outpatient		
	Non-VHA (n = 52)	Non-VHA (n = 133)	VHA (n = 55)	p Value^†^
	Count (%)	Count (%)	Count (%)	
Primary EMR system				
Cerner	14 (26.9%)	13 (9.8%)	2 (3.6%)	< 0.001
Epic	27 (51.9%)	96 (72.2%)	0 (0.0%)	
Other	11 (21.2%)	24 (18%)	53 (96.4%)	
Other work environment characteristics				
Uses additional AC management software	13 (25.0%)	43 (32.3%)	46 (83.6%)	< 0.001
Has organized service to manage warfarin	47 (90.4%)	129 (97%)	54 (98.2%)	NS
Has organized service to manage DOACs	30 (57.7%)	97 (72.9%)	54 (98.2%)	< 0.001
Reports having dashboard for warfarin management	16 (30.8%)	45 (33.8%)	24 (43.6%)	0.20
Reports having dashboard for DOAC management	11 (21.2%)	28 (21.1%)	54 (98.2%)	< 0.001
Can display multiple warfarin users on demand	30 (57.7%)	67 (50.4%)	30 (54.5%)	NS
Can display multiple HRD users on demand	18 (34.6%)	19 (14.3%)	16 (29.1%)	0.002
Reported need for digital resources to enhance DOAC management				
Strongly agree	38 (73.1%)	91 (68.4%)	25 (45.5%)	0.018^‡^
Agree	13 (25.0%)	32 (24.1%)	20 (36.4%)	
Neutral	1 (1.9%)	9 (6.8%)	8 (14.5%)	
Disagree	0 (0%)	1 (0.8%)	2 (3.6%)	
Strongly disagree	0 (0%)	0 (0%)	0 (0.0%)	

^†^Statistical tests compared outpatient groups only

^‡^Statistical test performed across group of response options

*AC* anticoagulant, *DOAC* direct oral anticoagulant, *EMR* electronic medical record, *HRD* high risk drug (e.g., diabetes, opioids), *VHA* Veterans Health Administration

Among acute care respondents (all from non-VHA settings), the majority were again pharmacists (44/52, 84.6%; Table 1.). Respondents worked primarily in direct patient care roles (34/52, 65.4%) and reported practicing across a broad range of specialty areas. Epic was the most commonly used EMR in this setting (27/52, 51.9%; Table 2.). Although no statistical comparisons were made comparing inpatient responses to those of outpatient VHA sites, the inpatient sites reported numerically lower rates of access to additional software for AC management (13/52, 25.0% vs. 46/55, 83.6% respectively), having organized services for DOAC management (30/52, 57.7% vs. 54/55, 98.2% respectively), and having access to a dashboard for DOAC management (11/52, 21.2% vs. 54/55, 98.2% respectively). Acute care sites also reported a strong desire for digital resources to enhance DOAC management.

### DOAC dashboard capabilities

Among respondents from the outpatient setting, VHA respondents were significantly more likely to report the ability to display multiple DOAC users on-demand (53/55, 96.4% vs. 47/133, 35.3% respectively; Table 3.), a foundational feature of digital dashboards. Regarding their access to specific digital dashboard features, the VHA sites were significantly more likely to have access to a dashboard that incorporates common indications for oral anticoagulation (e.g., atrial fibrillation) and that displays laboratory parameters and patient characteristics integral to the selection and appropriate dosing of available DOACs (e.g., age and serum creatinine). VHA respondents were also significantly more likely to report the ability of their systems to automatically identify potential problem types commonly associated with DOAC utilization (e.g., inappropriate dose, out-of-range laboratory values) and to track interventions and produce related reports. There were no significant differences in the ability of VHA and non-VHA outpatient respondents to have a dashboard fully integrated into their workflows or to be alerted to patients with DOAC prescriptions on hold, scheduled for medical procedures, missing appointments, or lacking anticoagulation despite the presence of an indication for treatment (e.g., untreated atrial fibrillation). DOAC management dashboards at VHA outpatient sites were reported to be significantly more advanced, with the 49/55 (89.1%) being in regular clinical use for most eligible patients under their care (p < 0.001; Table 4.).

**Table 3:** DOAC dashboard capabilities

	Inpatient	Outpatient		
	Non-VHA (n = 52)	Non-VHA (n = 133)	VHA (n = 55)	p Value^†^
	Count (%)	Count (%)	Count (%)	
Patient identification and workflow integration				
Can display multiple DOAC users on demand* ^††^	25 (48.1%)	47 (35.3%)	53 (96.4%)	< 0.001
DOAC dashboard display integrated into EHR workflow**	18 (34.6%)	35 (26.3%)	20 (36.4%)	NS
DOAC user types included in dashboard**				
Atrial fibrillation^††^	19 (36.5%)	43 (32.3%)	53 (96.4%)	< 0.001
Venous thromboembolism^††^	19 (36.5%)	42 (31.6%)	53 (96.4%)	< 0.001
CAD/PAD^††^	13 (25%)	24 (18.0%)	18 (32.7%)	0.045
Heart valves^††^	16 (30.8%)	25 (18.8%)	36 (65.5%)	< 0.001
Key data fields displayed within DOAC dashboard**				
Indication for DOAC^††^	7 (13.5%)	22 (16.5%)	53 (96.4%)	< 0.001
Weight^††^	21 (40.4%)	23 (17.3%)	51 (92.7%)	< 0.001
Age^††^	25 (48.1%)	34 (25.6%)	51 (92.7%)	< 0.001
Sex^††^	24 (46.2%)	36 (27.1%)	40 (72.7%)	< 0.001
Serum creatinine^††^	20 (38.5%)	27 (20.3%)	53 (96.4%)	< 0.001
Creatinine clearance^††^	22 (42.3%)	22 (16.5%)	51 (92.7%)	< 0.001
Hemoglobin^††^	16 (30.8%)	15 (11.3%)	52 (94.5%)	< 0.001
Hematocrit	12 (23.1%)	11 (8.3%)	38 (69.1%)	< 0.001
Anticoagulant on hold	4 (7.7%)	12 (9.0%)	4 (7.3%)	NS
Scheduled procedure	1 (1.9%)	9 (6.8%)	3 (5.5%)	NS
Count of key data fields displayed in DOAC dashboard**				
0	27 (51.9%)	91 (68.4%)	2 (3.6%)	< 0.001^‡^
1–3	3 (5.8%)	12 (9.0%)	0 (0%)	
4–6	10 (19.2%)	18 (13.5%)	9 (16.4%)	
7–10	12 (23.1%)	12 (9.0%)	44 (80.0%)	
Potential problems displayed within a DOAC dashboard**				
Inappropriate drug^††^	1 (1.9%)	7 (5.3%)	34 (61.8%)	< 0.001
Inappropriate dose^††^	4 (7.7%)	9 (6.8%)	53 (96.4%)	< 0.001
Missing/Out of range lab value^††^	7 (13.5%)	12 (9.0%)	53 (96.4%)	< 0.001
Patient non-adherence^††^	2 (3.8%)	8 (6.0%)	50 (90.9%)	< 0.001
Patient missed appointment	2 (3.8%)	9 (6.8%)	2 (3.6%)	NS
None of potential problems displayed	42 (80.8%)	112 (84.2%)	2 (3.6%)	< 0.001
Additional features available within a DOAC dashboard**				
Tracks clinician interventions^††^	10 (19.2%)	11 (8.3%)	40 (72.7%)	< 0.001
Produce DOAC-related reports^††^	4 (7.7%)	11 (8.3%)	28 (50.9%)	< 0.001
Identifies patients eligible for AC but not currently prescribed	3 (5.8%)	3 (2.3%)	4 (7.3%)	NS

*Ability to generate a multi-patient DOAC user roster on-demand was considered a foundational capability upon which all other dashboard features and capabilities are contingent

**Questions posed only to respondents who affirmed ability to generate a DOAC user roster on-demand, with “no” response being imputed to all other respondents without roster-generating ability

^†^Statistical tests compared outpatient groups only

^‡^Statistical test performed across group of response options

^††^Indicates feature known to be available in VHA DOAC dashboard

*AC* anticoagulant, *CAD/PAD* coronary artery disease/peripheral artery disease, *DOAC* direct oral anticoagulant, *EHR* electronic health record, *VHA* Veterans Health Administration

**Table 4:** Development stage of DOAC dashboard

	Inpatient	Outpatient		
	Non-VHA (n = 52)	Non-VHA (n = 133)	VHA (n = 55)	
	Count (%)	Count (%)	Count (%)	p Value^†^
Development stage of DOAC dashboard				
Initial development/testing	5 (9.6%)	12 (9.0%)	2 (3.6%)	< 0.001^‡^
Limited pilot use underway	2 (3.8%)	5 (3.8%)	0 0.0%)	
Regular clinical use for subset	5 (9.6%)	10 (7.5%)	2 (3.6%)	
Regular clinical use for most eligible patients	9 (17.3%)	13 (9.8%)	49 (89.1%)	
None or Other	31 (59.6%)	93 (69.9%)	2 (3.6%)	

^†^Statistical tests compared outpatient groups only

^‡^Statistical test performed across group of response options

*DOAC* direct oral anticoagulant, *VHA* veterans health administration

Inpatient respondents were numerically less likely to have the ability to display multiple DOAC users on demand than VHA outpatient sites (25/52, 48.1% vs. 53/55, 96.4% respectively; Table 3.). The inpatient respondents were also numerically less likely to have access to a dashboard that incorporates indications for oral anticoagulation, displays laboratory parameters or patient characteristics integral to the selection and appropriate dosing of DOACs, or to identify problems commonly associated with DOAC utilization. Fewer than 8% of inpatient respondents reported access to a dashboard with the ability to display patients with anticoagulants on hold, with scheduled invasive procedures, missed appointments, or with untreated indications for anticoagulation. Only a minority of inpatient respondents reported access to a digital dashboard that tracks anticoagulation-related interventions or has the ability to generate related reports. Dashboards that were described by inpatient sites varied widely in their stages of development, with only 9/52 (17.3%) being in regular clinical use for most eligible patients.

### Expert panel

The 10 authors and 14 additional anticoagulation specialists, informaticists, and clinicians with clinical dashboard implementation expertise for anticoagulant or other high-risk drug classes convened for a 3-h remote discussion. Moderated by authors with expertise in DOAC dashboard implementation (AA, GB), robust discussions generated several key findings. Two key themes were identified: vision for dashboard capabilities and barriers to development and adoption.

### Vision for dashboard capabilities

Experts recommended: (a) focusing on the core functional domains described by others (Fig. 1.); (b) desire for customizability (or multiplicity of available packages); (c) incorporation into existing workflows and portability; (d) desire for the inclusion of untreated patients appropriate for prophylaxis or treatment with anticoagulants; (e) desire for reports that capture and articulate operational efficiencies and fiscal impact.

### Barriers to development and adoption

The most important barrier to the development and implementation of DOAC dashboards identified by the experts was the lack of a regulatory mandate for Anticoagulation Stewardship. Antimicrobial Stewardship has been established as a Condition of Participation for Medicare and Medicaid programs [19] and, as a result, EHR vendors have developed and marketed digital resources (including dashboards) to support health systems in that area. To date, Anticoagulation Stewardship is not mandated for health systems, so digital tool development has not been prioritized by the industry due to factors such as competing priorities and resource scarcity.

Additionally, global vendors may limit the depth and variety of features so that their systems have the broadest possibly utility. Because no single digital resource is likely to satisfy all use cases, multiple EHR and secondary vendor products are likely needed to accommodate different clinical implementation models (e.g., centralized population management vs. traditional patient-clinician encounters). Lastly, data quality, tool usability, and perceived value to end users (e.g., workflow efficiency) and system leadership were identified as factors that may affect uptake and sustainability of dashboards upon implementation.

## Discussion

This initiative presents diverse perspectives and insights into the status of digital dashboards in clinical practice and the barriers that impede their widespread adoption for anticoagulation management. The survey, completed primarily by pharmacists practicing clinically in anticoagulation management, suggests that digital dashboards for DOAC management are not yet widely available to clinicians in the United States apart from outpatient clinics within the VHA. Clinics within the VHA were significantly more likely to report having a formal service for managing DOACs and approximately five times more likely to have access to a digital dashboard to manage these agents. Respondents from VHA clinics consistently reported the capacity to identify DOAC users, display key clinical data fields, and alert clinicians to common DOAC-related problems, whereas non-VHA clinics were significantly less likely to do so. The responses from VHA sites are consistent with what is known about the VHA DOAC population health management tool through reports from the agency and are indicative of a robust, mature digital resource that has been successfully implemented and scaled at the national level [11–16] Responses from non-VHA clinics and hospitals suggest a degree of organic growth of such digital resources, but lacking the consistency and maturity of the VHA product.

The ACF survey and expert panel highlighted several key limitations and potential areas for growth in developing and implementing EHR-based dashboards to improve safe DOAC prescribing. The survey highlighted key differences in the ability for anticoagulation staff to identify treated patients, collect data on key patient characteristics that influence appropriate prescribing, and flag patients at highest risk for inappropriate DOAC use. The expert panel highlighted highly desired features and important barriers to the developing and broad update of an EHR-enabled dashboard for DOAC prescribing.

While health systems such as the VHA and the University of Michigan have successfully implemented digital dashboards to help manage large populations of patients prescribed DOACs [11–16], this survey of ACF members demonstrates that most inpatient and outpatient respondents do not yet have the ability to generate a current active roster of patients utilizing DOACs, present key clinical data elements, identify common DOAC-related problems, or facilitate clinical interventions or quality reporting. While some inpatient facilities appear capable of generating patient rosters with some relevant clinical data (most likely, “pursuit lists” to guide manual review of individual DOAC user profiles), they appear unable to use technology to proactively identify common problem types, prioritize patient cases, or facilitate clinician intervention. Respondents from non-VHA outpatient sites also lack access to the tools and processes now nearly universally available to clinicians across the country within the VHA system. This is disappointing, considering the fact that 72.9% reported having organized clinical services for DOAC management and that the VHA has already demonstrated the ability to improve prescribing practices and make clinical intervention more efficient.

Although the VHA has the benefit of a single EHR platform and a relatively closed health system, the data elements utilized in their DOAC dashboard (i.e., patient characteristics, drug profiles, common laboratory test results) are standard components of available EHRs. Likewise, the logic used to identify potential problems is aligned with FDA package labeling and has been published as an appendix to a prior VHA manuscript [14]. Further, the development and implementation of the dashboard in Michigan proves that the approach is feasible in the private sector, supporting the premise that the development and broad dissemination of EHR-based DOAC management dashboards is hindered by regulatory and administrative barriers, not technical feasibility.

The findings of the current survey illustrate the advances that the VHA has made in the development and implementation of their dashboard and the considerable room for improvement among non-VHA outpatient sites that do not yet have access to this technology. The responses may also illustrate room for improvement with the VHA tool and its implementation, as it does not yet appear to be fully integrated into clinician workflows and does not yet identify prescribed anticoagulants that are on hold for scheduled invasive procedures, or patient missed appointments. Because the VHA dashboard is currently implemented to support pharmacy operations, it does not currently display patients with indications for anticoagulation that are not being prescribed treatment, an important population health and quality of care consideration for that health system.

### Strengths and weaknesses

The current work has several notable strengths. The efforts were led by ACF, a well-respected non-profit organization with deep experience in Anticoagulation Stewardship, and all aspects of the work were conducted in a brand-agnostic manner. The survey recipients were active members of ACF, suggesting interest and knowledge in the field of interest. The survey instrument was designed to assure data integrity, and analysis was limited to complete responses. While the overall response rate was modest (8.5%), this rate is in line with figures reported for email surveys of this nature [20] and the final number of evaluable responses (240) from across care settings was sufficient to illustrate significant differences between outpatient VHA sites (where dashboard use is known) and non-VHA clinics. Lastly, responses from VHA outpatient sites (where system characteristics are known), were highly consistent, supporting the validity of the survey instrument.

The effort is also characterized by inherent weaknesses that may impact upon the results or limit their generalizability. Survey recipients were all members of ACF, potentially limiting the generalizability of the result to the broader US health system. Although ACF is a multidisciplinary organization, its membership is known to be primarily pharmacists in the outpatient setting, possibly impacting the generalizability further. The decision to respond may have been influenced by the recipient’s own interests and experiences in this area, potentially biasing the results. The low response rate (8.5%) may introduce meaningful selection bias and, because recipients may utilize personal or work email addresses, it was not possible to identify or evaluate multiple responses from a single health system. Furthermore, a lack of data describing ACF members limits our ability to compare respondents and non-respondents.

Regarding the expert forum, opinions were solicited from a convenience sample of participants, and not all major US primary EHR vendors or secondary health care software vendors were present. The proprietary nature of the industry may also have limited dialogue and sharing among those who did attend. Despite these limitations, the authors believe that the findings do add meaningful insights into the state of DOAC management practices and dashboard development. Further, the survey instrument (either in its current form or with modifications) may be useful in future efforts targeting more representative recipient pools to better track the evolution of dashboards in managing this growing population of high-risk drug users.

## Conclusion

Although digital DOAC dashboards are technically feasible and have been successfully implemented to support the clinical management of select outpatient populations and to advance Anticoagulation Stewardship efforts, they have not been broadly adopted as a model of care beyond outpatient services within the VHA system. Regulatory requirements and other incentives are likely necessary for DOAC dashboards and similar EHR-based tools to be adopted widely in the US. Research demonstrating a significant impact on clinical and fiscal outcomes is needed to influence health policy and administrative decision-making in this regard.

### Supplementary Information

Below is the link to the electronic supplementary material.

Supplementary file1 [Survey Instrument] (PDF 241 kb)

Supplementary file2 [Expert Panel Description] (DOCX 16 kb)

## Data Availability

Survey data will be made available upon request.
