# Supplementary material for: Digital dashboards for direct oral anticoagulant surveillance, intervention and operational efficiency: uptake, obstacles, and opportunities
Source: J Thromb Thrombolysis. 2023 Oct 15;57(1):107–16. doi: 10.1007/s11239-023-02893-9 (PMC10830621; doi:10.1007/s11239-023-02893-9)
Supplement: Supplementary file 1 — Supplementary file1 [Survey Instrument] (PDF 241 kb) [file 11239_2023_2893_MOESM1_ESM.pdf]

Anticoagulation FORUM

Anticoagulation Forum Survey: Systematic DOAC Management and Digital "Dashboards"

Dear Anticoagulation Forum Member,

Thank you for taking the time to complete this brief survey (~10 mins).

We know that direct oral anticoagulants (DOACs) are now prescribed more frequently than warfarin. Now we want to know whether 1) health systems have implemented processes for the systematic clinical management of DOACs and 2) if digital population management resources (e.g., "dashboards") are being utilized to support such systematic management.

This survey seeks to ascertain the prevalence of systematic DOAC management and the degree to which digital "dashboards" are currently in use to support such management.

You will not need any other resources to complete the survey. Results will be de-identified, aggregated and utilized to guide discussions at an upcoming expert forum and in a manuscript submitted to a peer-reviewed medical journal.

The Anticoagulation Forum may ask additional questions to respondents who describe advanced experiences with DOAC management and digital dashboards. However, further participation is completely voluntary.

Optional: Individuals who complete the survey will be entered into a lottery to receive one of 6 complementary admissions to either the 2022 October Virtual Boot Camp (October 7-8, 2022) or the Anticoagulation Forum's 17th National Conference on Anticoagulation Therapy (April 1-3, 2023 in San Francisco, CA). See entry details at the end of the survey!

This survey is supported in part by funding from Pfizer Inc.

Anticoagulation FORUM

Anticoagulation Forum Survey: Systematic DOAC Management and Digital "Dashboards"

Respondent Characteristics

Please select the responses that best describe your primary role and the primary care setting in which you work or practice clinically (i.e., the perspective from which you will complete the survey)

* 1. First Name:

* 2. Last Name:

* 3. Email:

* 4. Clinical Organization Name:

* 5. Profession:

Anticoagulation FORUM

Anticoagulation Forum Survey: Systematic DOAC Management and Digital "Dashboards"

* 6. What is the main EHR system used in your primary work setting?

- ○ Epic
- ○ Cerner
- ○ Allscripts
- ○ Meditech
- ○ Athena
- ○ Other (please specify)

* 7. Does this site utilize additional software or digital platforms to support the clinical management of patients utilizing anticoagulants?

- ○ Yes
- ○ No
- ○ Unknown

Anticoagulation
FORUM

Anticoagulation Forum Survey: Systematic DOAC Management and Digital "Dashboards"

8. If your site uses additional software or digital platforms to support the clinical management of patients utilizing anticoagulants, please provide the name and a brief description of how it is used:

Anticoagulation
FORUM

Anticoagulation Forum Survey:
Systematic DOAC Management and Digital "Dashboards"

Systematic Management of Warfarin, DOACs

* 9. My primary practice site has an organized service that provides clinical management to patients prescribed **warfarin**:

◯ Yes ◯ No ◯ Unknown

* 10. My primary practice site has an organized service that provides clinical management to patients prescribed **DOACs**:

◯ Yes ◯ No ◯ Unknown

Anticoagulation Forum Survey:
Systematic DOAC Management and Digital "Dashboards"

Population Health Management Tool (Dashboard) Definition

Please stop, hit play, and listen to the audio on the following video before proceeding with the survey

Use expand feature on video to see larger figure. Once the video is complete, please exit full screen mode to return to the survey.

For this survey the definition of a digital "dashboard" DOES NOT include the following:

- Patient facing website or resources
- Clinical decision support features for single patients
- Retrospective quality reports or displays for managers

Anticoagulation
FORUM

Anticoagulation Forum Survey:
Systematic DOAC Management and Digital "Dashboards"

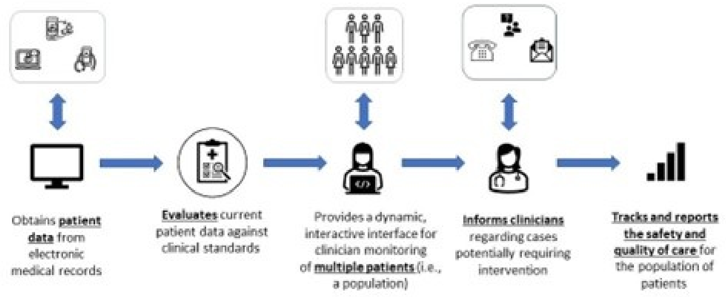

Site-Specific Questions:

* 11. Considering the definition above, my primary work site HAS a digital population health management dashboard available for managing patients prescribed warfarin:

◯ Yes ◯ No ◯ Unknown

* 12. Considering the definition above, my primary practice site HAS a digital population health management dashboard available for managing patients prescribed DOACs:

◯ Yes ◯ No ◯ Unknown

* 13. With regard to my primary work setting, and without additional IT work, I can utilize an electronic system to simultaneously display the identities of multiple patients currently prescribed DOACs:

◯ Yes ◯ No ◯ Unknown

Anticoagulation
FORUM

Anticoagulation Forum Survey: Systematic DOAC Management and Digital "Dashboards"

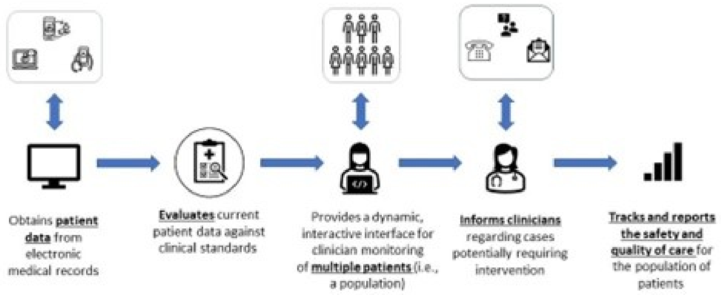

Features/Capabilities:

* 14. Is this display integrated into the main EHR and workflow (i.e., is accessible without additional login, use of separate software)?

◯ Yes ◯ No ◯ Unknown

* 15. Does the existing display include or access additional data fields beyond patient identifiers? (select all that apply)

- ☐ Indication for anticoagulation
- ☐ Patient weight
- ☐ Patient age
- ☐ Patient sex
- ☐ Others (please specify)
- ☐ Serum creatinine
- ☐ Creatinine clearance
- ☐ Hemoglobin
- ☐ Hematocrit
- ☐ Anticoagulant on hold
- ☐ Scheduled upcoming procedure/surgery
- ☐ None

* 16. Does the resource automatically identify potential problems with DOAC-related care (select all that apply):

- [ ] Inappropriate drug
- [ ] Missing/out of range lab value
- [ ] Patient missed appointment
- [ ] Inappropriate dose
- [ ] Patient non-adherence
- [ ] None
- [ ] Others (please specify)

* 17. Does the display facilitate and track the status of clinician interventions to address identified problems?

- ○ Yes
- ○ No
- ○ Unknown

* 18. Does the display have the ability to produce reports or graphics characterizing the quality of DOAC-related care provided by the clinic or service over time (i.e., performance across multiple patients)?

- ○ Yes
- ○ No
- ○ Unknown

* 19. Does the resource include or identify patients potential eligible for anticoagulation for whom it has not been prescribed (e.g., untreated atrial fibrillation)?

- ○ Yes
- ○ No
- ○ Unknown

20. Please provide any additional comments about the features and capabilities of the digital DOAC management resource at your primary practice site:

Anticoagulation FORUM

Anticoagulation Forum Survey: Systematic DOAC Management and Digital "Dashboards"

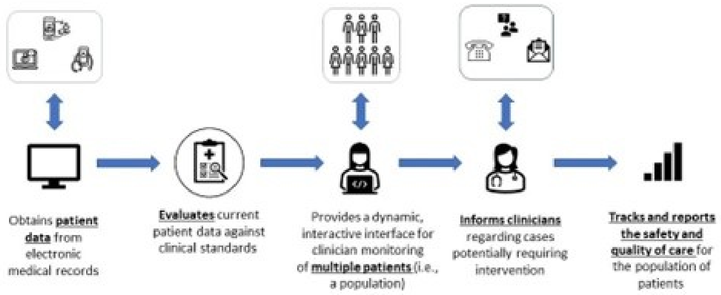

Operations and Workflows:

* 21. Which clinical service(s) are expected to utilize the DOAC display as part of the patient care process? (check all that apply)

- ☐ Pharmacy
- ☐ Advanced Practices Nursing
- ☐ Physician Assistant
- ☐ Physicians
- ☐ Nursing
- ☐ Other (please specify)

* 22. How frequently is this display typically reviewed? [Select option that best describes use of the dashboard at your site]

- ◯ < Once per week
- ◯ Once per week
- ◯ 2-4 times a week
- ◯ 5 times a week
- ◯ 7 times a week
- ◯ > 7 times per week
- ◯ Other (please specify)

* 23. Patients with which of the following conditions are included in the digital displays (select all that apply):

- [ ] Atrial fibrillation
- [ ] CAD/PAD
- [ ] VTE
- [ ] Heart valves
- [ ] Other (please specify)

* 24. At what stage of development/maturity would you say that your DOAC digital tool is currently functioning?

25. Please provide any additional comments about the operations and workflows involving digital tools to manage DOACs at your primary practice site:

* 26. With regard to my primary work setting, and without additional IT work, I can utilize an electronic system to simultaneously display the identities of multiple patients currently prescribed warfarin:

- ◯ Yes
- ◯ No
- ◯ Unknown

* 27. With regard to my primary work setting, and without additional IT work, I can utilize an electronic system to simultaneously display the identities of multiple patients currently prescribed other high-risk drugs (e.g., opioids, hypoglycemics):

- ◯ Yes
- ◯ No
- ◯ Unknown

* 28. I believe that there is need for improved digital tools to help clinicians better manage DOACs:

- ◯ Strongly agree
- ◯ Agree
- ◯ Neutral
- ◯ Disagree
- ◯ Strongly disagree

Anticoagulation
FORUM

Anticoagulation Forum Survey: Systematic DOAC Management and Digital "Dashboards"

Next Steps

Thank you for taking the time to complete this survey. Anticoagulation Forum also intends to reach out directly to select survey respondents to discuss oral anticoagulation management practices at their practice sites. If you are willing to communicate with us further regarding this topic, please indicate such by responding below.

* 29. Primary work setting:

○ Inpatient Care ○ Outpatient Care ○ Other Clinical Care Setting ○ Non-clinical Setting

Anticoagulation FORUM

Anticoagulation Forum Survey: Systematic DOAC Management and Digital "Dashboards"

* 30. Select any/all items that characterize your primary practice setting:

☐ Anticoagulation Clinic (general)
☐ Cardiology Specialty Clinic
☐ Hematology Clinic
☐ Other (please specify)

[ ]

Anticoagulation FORUM

Anticoagulation Forum Survey: Systematic DOAC Management and Digital "Dashboards"

* 31. Select any/all items that characterize where you work within your primary practice setting:

- [ ] Emergency Department
- [ ] Critical Care
- [ ] Surgery
- [ ] Adult Internal Medicine
- [ ] Neurology
- [ ] Anticoagulation Stewardship
- [ ] Cardiology
- [ ] Hematology
- [ ] N/A
- [ ] Other (please specify)

Anticoagulation FORUM

Anticoagulation Forum Survey: Systematic DOAC Management and Digital "Dashboards"

* 32. What is your primary role in that primary setting?

- ◯ Direct Patient Care Clinician
- ◯ Clinic/Department Management
- ◯ Executive Leadership
- ◯ Other (please specify)

[ ]

Anticoagulation
FORUM

Anticoagulation Forum Survey: Systematic DOAC Management and Digital "Dashboards"

* 33. Is your primary work setting an Anticoagulation Forum Center of Excellence?

◯ Yes ◯ No ◯ Unknown

* 34. I am interested in speaking with Anticoagulation Forum further regarding our oral anticoagulation management practices

◯ Yes ◯ No

* 35. Lastly, to express our gratitude for the responses we've received to the survey, Anticoagulation Forum is entering the names of interested survey respondents into a raffle of 6 complementary registrations to either the 2022 October Virtual Boot Camp (October 7-8) or the Anticoagulation Forum's 17th National Conference on Anticoagulation Therapy (April 1-3, 2023 in San Francisco, CA). If you would like to be considered for the raffle, please indicate such by responding below:

◯ Yes, I would like to be included in the raffle of free registration

◯ No, I would not like to be included in the raffle of free registration

36. Please add any additional information on population health-based digital tools, and please feel free to add general comments for the AC Forum:

[ ]
