# Supplementary material for: Digital dashboards for direct oral anticoagulant surveillance, intervention and operational efficiency: uptake, obstacles, and opportunities
Source: J Thromb Thrombolysis. 2023 Oct 15;57(1):107–16. doi: 10.1007/s11239-023-02893-9 (PMC10830621; doi:10.1007/s11239-023-02893-9)
Supplement: Supplementary file 2 — Supplementary file2 [Expert Panel Description] (DOCX 16 kb) [file 11239_2023_2893_MOESM2_ESM.docx]

**Appendix B. Supplemental Information**

**Survey Distribution:**

Anticoagulation Forum is a non-profit, non-dues-bearing, multidisciplinary organization of anticoagulation management clinicians. The organization’s primary mission is the development and dissemination of educational resources and clinical tools relating to the prevention and treatment of thrombosis and bleeding. The survey was disseminated to the individuals in the United States who had participated in an Anticoagulation Forum-hosted webinar, conference, or bootcamp in the prior 24 months (n = 4,023). It was first disseminated on September 8, 2022, followed by a single reminder notification. Responses received before September 26, 2022, were included in the analysis.

**Expert Forum:**

A group of experts in clinical management of anticoagulants and in the development and implementation of digital resources to manage high-risk drugs (e.g., anticoagulants, opioids, and others) was convened virtually via the Zoom™ platform. This group discussed benefits and features of digital population health management tools, explored successful implementation strategies and experiences, and identified barriers to their widespread development and adoption across care settings for oral anticoagulants. Participants were identified through the ACF Board of Directors, authors of relevant manuscripts identified in the literature search, and through professional relationships of the authors.

The participant list was intended to represent a balance of anticoagulation management specialists and subject matter experts in either health informatics (e.g., developers) or clinicians with experience in dashboard implementation. During the event, participants were oriented to anticoagulants as national public health priority, anticoagulation stewardship, national data on anticoagulant-related harms, findings of the literature search regarding DOAC dashboards, and preliminary results of the ACF member survey. Discussion then ensued around desired DOAC dashboard features and barriers to advancing the development and uptake of digital tools to better manage DOACs in clinical practice. Key themes were documented from the discourse and distilled into qualitative findings for each theme (i.e., vision for dashboard capabilities, barriers to development and adoption).

**Description of Contributors to Expert Forum**

The Expert Forum included guest participants with the following backgrounds and perspectives:

Academic cardiologist with leadership role in a health system’s clinical informatics department

Associate Chief Medical Informatics Officer for a major academic medical center

Chief Operating Officer of a software development firm utilizing artificial intelligence

Chief of Pharmacy at a major academic medical center

Director of Education for a nonprofit organization in health care quality and safety realm

Medical Director of quality at a major academic medical center

Medical informaticist and certified builder from a major academic medical center

Pharmacist informaticist with a leadership role within a US government health system

Pharmacy Manager from a major academic medical center

Senior executive pharmacist from a US government health system

Senior Product Manager for a major global electronic health record system

Vice President of Product Management at a large health system software company.
